# Compartmentalized microchannel array for high-throughput analysis of single cell polarized growth and dynamics

**DOI:** 10.1038/srep16111

**Published:** 2015-11-04

**Authors:** Tao Geng, Erin L. Bredeweg, Craig J. Szymanski, Bingwen Liu, Scott E. Baker, Galya Orr, James E. Evans, Ryan T. Kelly

**Affiliations:** 1 Environmental Molecular Sciences Laboratory, Pacific Northwest National Laboratory, Richland, WA, USA

## Abstract

Interrogating polarized growth is technologically challenging due to extensive cellular branching and uncontrollable environmental conditions in conventional assays. Here we present a robust and high-performance microfluidic system that enables observations of polarized growth with enhanced temporal and spatial control over prolonged periods. The system has built-in tunability and versatility to accommodate a variety of scientific applications requiring precisely controlled environments. Using the model filamentous fungus, *Neurospora crassa*, our microfluidic system enabled direct visualization and analysis of cellular heterogeneity in a clonal fungal cell population, nuclear distribution and dynamics at the subhyphal level, and quantitative dynamics of gene expression with single hyphal compartment resolution in response to carbon source starvation and exchange. Although the microfluidic device is demonstrated on filamentous fungi, the technology is immediately extensible to a wide array of other biosystems that exhibit similar polarized cell growth, with applications ranging from bioenergy production to human health.

The establishment of polarity is a critical cellular process to the development of diverse organisms^1^,^2^,^3^. The most extremely polarized cell growth is continuous tubular extension of a cell surface by adding new materials to the apical region of the cell^3^,^4^. A variety of cells including filamentous fungi, plant pollen tubes, root hairs, algal rhizoids and neurons exhibit this type of tip growth pattern. A hypha, which morphologically presents a long tube with a slightly tapered tip, functions as the fundamental unit of a filamentous fungus to perceive and react to various signals to regulate fungal growth, development and propagation^1^,^3^. In-depth understanding of the hyphae would not only be of great interest in elucidating fungal behavior and molecular regulation, but also lead to broad applications in biotechnology and bioenergy production^5^,^6^.

Previous studies on single hyphae have primarily relied on randomly loading fungal spores (conidia) onto a glass substrate or an agar block and then visualizing under a microscope^7^,^8^,^9^,^10^,^11^. These methods, however, lack temporal and spatial control over the tracking of hyphae due to extensive hyphal branching and incompatibility with long-term culture. Such measurements are thus limited to the observation of either young hyphae in early stages of growth^7^,^8^,^9^ or hyphae at the periphery of fungal colonies^10^,^11^, and fail to obtain information on the time-dependent changes over prolonged periods. Creating a controllable growth environment surrounding a hypha can also be difficult with conventional imaging platforms. The crude local environment results in low accuracy measurements, especially for monitoring cellular response to external cues. Additionally, it is challenging to manipulate and monitor several hyphae simultaneously in unconstrained environments, as their growth paths commonly intersect, which increases experimental complexity and time and also perturbs local environments around each cell. To quantify dynamics of a large cell population and understand cell-to-cell variability, a new approach is needed to confine many hyphae along parallel paths with controlled microenvironments.

Microfluidics is a powerful and prominent tool for manipulating and analyzing single cells owing to its ability to confine cells within microstructures having comparable dimensions to those of the cells^12^,^13^,^14^. The microfluidic chips enable well-defined microenvironments, high-throughput or parallelized manipulation, high level of integration and automation, and low consumption of reagents. Combined with advanced imaging technologies, a variety of microfluidic cell trapping and culture systems have been developed for measuring cell proliferation and fate, gene expression, and intracellular signaling pathways of mammalian cells^15^,^16^,^17^,^18^, yeasts^19^,^20^,^21^,^22^,^23^ and bacteria^19^,^24^,^25^,^26^ at the single cell level. However, reports on filamentous fungi in microfluidic devices have been scarce. The Nicolau group constructed mazelike microfluidic networks to mimic the natural habitat of filamentous fungi, and explored fungal cell behavior under geometric guidance^27^,^28^. Stanley *et al*.^29^ employed a microfluidic platform to investigate the interactions of filamentous fungi with bacteria. More recently, microfluidic systems have been developed for culturing tip-growing cells including neurons^30^,^31^ and plant pollen tubes^32^. Nevertheless, these devices lack high-throughput capabilities and efficient compartmentalization of single cells for long-term tracking.

Here we present a high-performance microfluidic platform to trap single conidia and grow single hyphae in spatially separated channels for comparative hyphal growth and dynamics analysis. The device features 50 parallelized single-cell trapping sites and growth channels isolated by a single pneumatic microvalve as well as a common channel for microvalve-guided infusion of media, buffers, reagents, etc., allowing direct visualization of hyphal growth in a dynamically controlled yet shear stress-free microenvironment. Each conidium is trapped at the entrance of long, narrow growth channels of tunable dimensions, allowing individual hypha confinement throughout the experiments. This layout enables different filaments to be imaged in rapid succession by simply translating the microscope stage along one dimension. The functionality of the device is demonstrated with *Neurospora crassa*, a model organism for studying polarized cell growth and filamentous fungal biology^33^. Coupled with live-cell imaging and fluorescent fusion protein technologies, we have probed single hypha growth kinetics, nuclear organization and migration, and long-term gene expression dynamics in response to carbon source switching.

## Results

### Microfluidic Device Design

**Figure 1:**
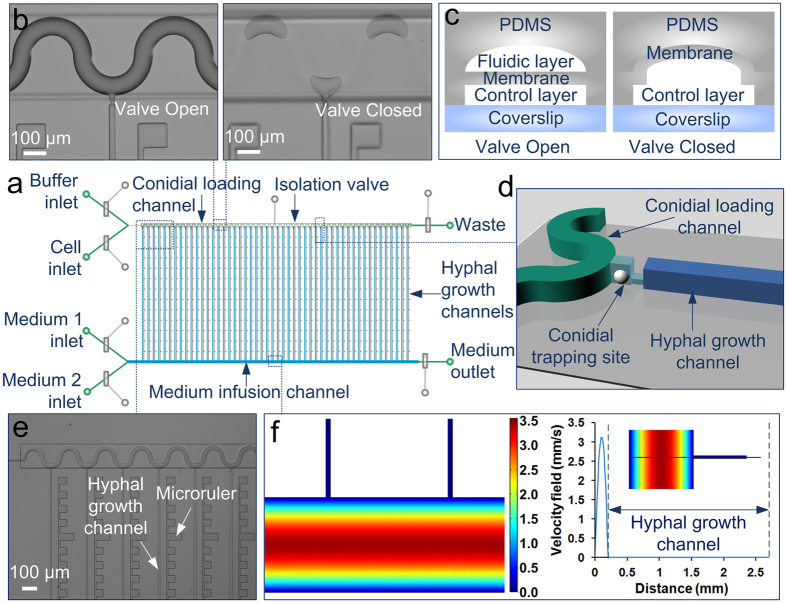
Microfluidic channel array for analyzing hyphal growth and dynamics. (**a**) Schematic of the chip layout comprising two layers of channels: a fluidic layer (green, light blue, and blue) and a control layer (grey) with 7 pneumatic microvalves. The fluidic layer consists of four inlets to deliver buffer, cells, and two types of medium, respectively, two outlets, a conidial loading channel (green), a medium infusion channel (blue), and 50 hyphal growth channels (blue) arrayed in parallel. (**b**) Image of the serpentine conidial loading channel precisely aligned with the isolation valve. The closure of the valve can compartmentalize each growth channel. Scale bar, 100 μm. (**c**) Schematic of the cross section of a valve. The fluidic channels having rounded cross sections (green) are completely closed when the valves are actuated. **(d)** Schematic of a conidial trapping site containing two short channels (light blue) with different dimensions and connecting the conidial loading channel (green) and the hyphal growth channel (blue). A single conidium could be captured by the constricted shallow channel. **(e)** A micrograph showing the channels and the microrulers at the right side of the hyphal growth channels. Scale bar, 100 μm. **(f)** Numerical simulation of flow profiles in the channels using COMSOL Multiphysics software. The right panel shows the velocity along the cross line from the bottom of the medium infusion channel to the top of the hyphal growth channel (as inset). No flow is present in the hyphal growth channels.

The microfluidic device was constructed from polydimethylsiloxane (PDMS) using multilayer soft lithography to enable flexible fluidic flow control through pneumatic microvalves (Fig. 1). It features two layers of channels: (1) fluidic channels (green, light blue and blue) for cell/reagent transport and manipulation on top; and (2) 25-μm-high control channels (grey) for valve actuation on bottom. The two layers were separated by a 50-μm-thick PDMS membrane. The fluidic layer could be generally divided into two major areas (Fig. 1a). The first area is composed of four inlets to deliver buffer, cells, and two types of media, respectively, two outlets for media and waste, and sample delivery channels close to the inlets and outlets (green). The second area is the main functional area comprising a serpentine conidial loading channel (40 μm wide × 10 μm high, green) (Fig. 1b), a medium infusion channel (200 μm wide × 10 μm high, green), and 50 hyphal growth channels (blue) arrayed in parallel, each with a dimension of 2.5 mm × 10 μm × 10 μm (length × width × height) if not specified. In addition, an array of 50 conidial trapping sites (light blue), each comprising two short channels with different dimensions (8 μm × 8 μm × 10 μm and 10 μm × 6 μm × 2 μm), is located between the conidial loading channel and the hyphal growth channels (Fig. 1d). The channels shown in green have rounded cross sections to allow complete closure when the valves were actuated (Fig. 1c). Microrulers with no fluidic connectivity are patterned between hyphal growth channels to facilitate length measurements (Fig. 1e). The number of parallel growth channels can be easily decreased or increased to meet experimental needs.

### Single-Conidium Trapping and Growth Conditions

**Figure 2:**
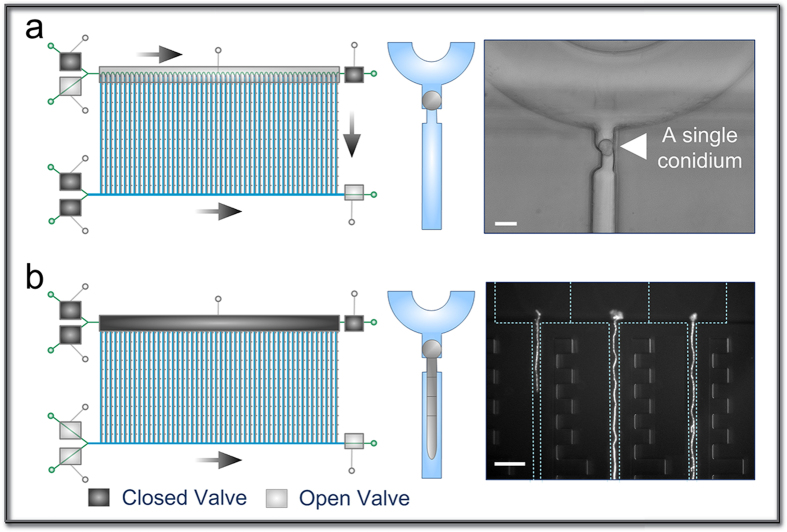
Single-conidium trapping and compartmentalized hyphal growth. **(a)** A single conidium of *N. Crassa* hydrodynamically trapped at the conidial trapping site. The conidia are loaded at a concentration of 2 × 10^5^ cells/mL in 1× Vogels’ salts and a flow rate of 0.4 μL/min, with the medium outlet valve open while the waste valve closed. Scale bar, 10 μm. **(b)** The compartmentalized hyphal extension of *N. Crassa* along the hyphal growth channels (2.5 mm × 10 μm × 10 μm) due to the closure of isolation valve. The hyphae are cultivated under constant flow of 10% glucose in 1× Vogels’ salts solution at 0.3 μL/min. Scale bar, 100 μm.

Due to the cylindrical shape of the hyphae, it is ideal to trap the conidium at the entrance of a microchannel into which a hypha can elongate. Thus, we used a hydrodynamic flow-assisted trapping mechanism to accomplish single-cell entrapment. Since the diameter of the *N. crassa* conidia is 3 to 8 μm, a single conidium could be hydrodynamically retained by the constriction structure due to the difference in the cross-sectional areas between the trapping channel and the cell when the media outlet valve was open and the waste outlet valve was closed (Fig. 2a). Once a trapping site was occupied, the serpentine loading channel caused subsequent conidia to preferentially travel to the next empty trapping structure. Using this method, we could achieve a trapping efficiency of ~70%. Although some sites accumulated multiple cells because of variations in cell dimension and shape, only a single hypha could extend into a hyphal growth channel due to the confinement of the shallow channels in the trapping sites. Remarkably, the conidial loading channel was large enough to allow free cell movement without clogging.

To induce conidial germination and hyphal extension, the isolation valve and the waste outlet valve were actuated, and medium was constantly perfused into the medium infusion channel (Fig. 2b). A unique benefit of our system is that the conidia could be compartmentalized by the actuation of the isolation valve to maintain each hypha separated from each other in different channels while still experiencing equivalent environments. This is owing to our ability to precisely align the microvalves to the fluidic channels of interest and fully close the major regions of the serpentine channel using the valve with width identical to the peak-to-peak distance of the channel (Fig. 1b). Such compartmentalization eliminated hyphal extension into the conidial loading channel and cross-contamination caused by intercellular interactions and hyphal fusion, and instead directed hyphal elongation only through the narrow channels towards the medium infusion channel, thereby allowing independent and accurate analysis of individual hyphae (Fig. 2b). It is worth noting that we utilized a single microvalve for compartmentalization, which greatly simplified the device design.

Our system also provided a stable and constant environment during the entire experiment, facilitating probing cell-to-cell viability under equivalent environments. Numerical simulation of the flow profile for the device reveals that there was no flow across the hyphal growth channels, since only one end of the long channel was exposed to fluidic flow while the other end was completely closed, and each channel exhibited a nearly identical profile (Fig. 1f). Nutrients were continuously transported into the hyphal growth channels *via* diffusion without disturbing the position and extension of the hyphae. The flow pattern created a shear stress-free environment for hyphal growth, which was essential for exploring cellular response to biochemical factors. The hyphal growth channels (10 μm high) were designed to be larger than the *N. crassa* hyphae, which had an average diameter of 7 μm, to ensure that medium exchange was not blocked. This was verified by introducing 100 μm of tracer dye 2-NBDG, a fluorescent analogue of glucose, into the device, and the channels filled with growing hyphae became fluorescent (Supplementary Fig. 1).

### Hyphal Growth and Morphology

**Figure 3:**
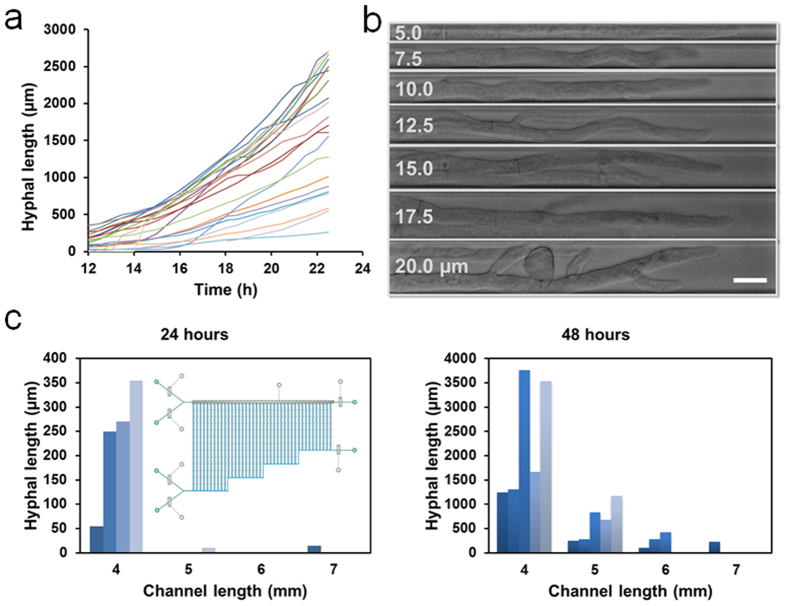
Hyphal growth and morphology on the microfluidic chips. **(a)** Growth curves of 22 individual hyphae growing in the channels (2.5 mm × 10 μm × 10 μm) on a single chip. The hyphal length is measured at regular intervals of 30 min starting 12 h after conidial trapping. **(b)** Images of hyphae growing in 7 different widths (5, 7.5, 10, 12.5, 15, 17.5 and 20 μm) of channels (2.5 mm long). Scale bar, 10 μm. **(c)** Hyphal extension in 4 different lengths (4, 5, 6 and 7 mm) of channels (10 μm wide) with 5 replicates for each length after 24 hours (left) and 48 hours (right). Inset shows a schematic of chip design. In the experiments, *N. Crassa* strain NMF617 expressing histone H1-RFP is grown under the constant flow of 10% glucose in 1× Vogels’ salts solution at 0.3 μL/min.

Previous studies of hyphal growth kinetics focused primarily on the measurement of total hyphal length of a mycelium and the hyphae in long horizontal glass tubes (“race tubes”) or at the margin of a colony^34^,^35^. With our system, we could accurately monitor the progressive extension of a single hypha germinated from a conidium over a long time and a long distance. We evaluated the hyphal extension of *N. crassa* strains in an array of 2.5 mm × 10 μm × 10 μm channels for 23 h (Supplementary Video 1). Figure 3a shows the lengths of 22 individual hyphae of histone H1-RFP strain NMF617 growing on a microchip as a function of time. In all cases, hyphal extension proceeded throughout the entire measurement period. However, the hyphae exhibited substantial cellular heterogeneity in terms of germination time and extension rate under equivalent growth conditions. Although 6 conidia started to develop after 12 h, the majority of the conidia germinated prior to that time. Moreover, the hyphae did not elongate at a constant rate, and the growth was slightly accelerated after they reached to ~500 μm, possibly because of less time required for nutrient diffusion. The fastest growing hyphae extended to the end of the 2.5-mm-long channel within 23 h, whereas the slowest growing hyphae was <300 μm long at that time. The typical observed growth rates of ~20–700 μm/h are in between those of germlings (~20 μm/h) and mature hyphae (~6 mm/h or more)^36^,^37^. Wild-type *N. crassa* strain exhibited similar growth patterns (Supplementary Fig. 2). Furthermore, the lower concentration (2% and 5%) of glucose reduced the growth rate (data not shown), and 10% glucose was used to ensure sufficient nutrient supply into the long channels. Notably, the extension rates were stochastic and independent of the channel location with no clear pattern from one experiment to the next, suggesting these observations are due to cell-to-cell variability in the clonal population.

The channel geometry had great impact on hyphal growth and morphogenesis. To assess the effect of confinement level, we cultivated *N. crassa* in a device with varying widths (5, 7.5, 10, 12.5, 15, 17.5 and 20 μm) of growth channels while keeping the length (5 mm) and depth (10 μm) constant (Fig. 3b). The hyphal extension rate did not show substantial change as expected, because the depth of the channel, which was larger than hyphal diameter, allowed free nutrient diffusion regardless of channel width. The hyphae growing in wider channels (12.5 to 20 μm) generated significant branching, which interfered with the visualization of the leading hyphae. In contrast, no branching was observed for hyphae growing in 5 μm channels, yet the hyphal morphology (particularly cell borders) was obscured as the hyphae occupied the entire channel width. Intermediate channel widths (7.5 μm and 10 μm) greatly inhibited branching frequency while providing a reasonable space for single hypha imaging with clear cell boundaries, thus the 10-μm-wide channels were used for subsequent experiments. We also examined the influence of channel length using a device having channels of different lengths (2, 3, 4, 5, 6, 7, 8, and 9 mm) but the same width and depth (both 10 μm). Computational simulation of time-dependent diffusion indicates that the glucose concentration in the growth channels showed decreased concentration as a function of increased channel length after 4 h of diffusion, and reached a steady state at the location of the cell-trapping site for the 2 mm channel (Supplementary Fig. 3a). Given enough time, the longer channels also ultimately reached steady state levels. The simulation was verified by the generation of fluorescence gradients after flowing 100 μm of 2-NBDG for 0.5 h and 1 h (Supplementary Fig. 3b). We further tested the hyphal elongation in channels with lengths of 4, 5, 6, and 7 mm, with 5 replicates for each length. Both germination efficiency and hyphal length were higher in shorter channels at either 24 h or 48 h because of less diffusion time in shorter channels (Fig. 3c). Taken together, these results highlight the ability of our design to establish a chemical concentration gradient by simply changing the lengths of growth channels and administrating only one type of solution into a microchip.

### Nuclear Organization and Migration

**Figure 4:**
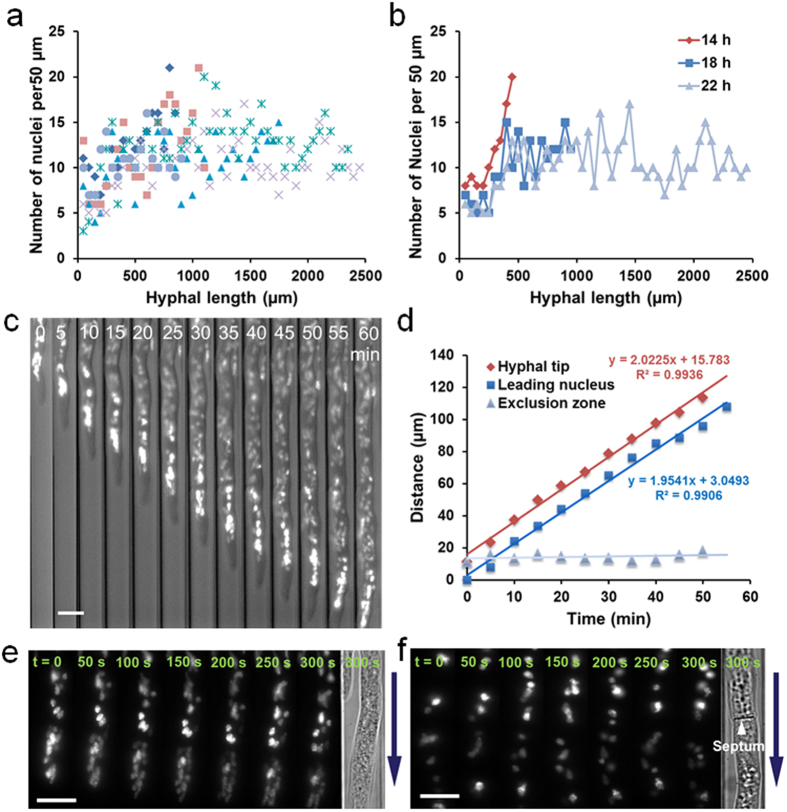
On-chip monitoring of nuclear organization and migration. **(a)** The nuclear distribution of 6 hyphae growing for 22 h on one microchip. The number of nuclei per unit length (50 μm) in hyphae is plotted vs. the hyphal length from the conidia to the tips. **(b)** A representative nuclear distribution dynamics of a hypha at 14, 18 and 22 h. The distribution slightly changes over hyphal extension. **(c)** Time-lapse imaging of long-range nuclear migration with combined bright-field and fluorescence microscopy over 60 min at intervals of 5 min. Scale bar, 10 μm. **(d)** Quantification of the extension of hyphal tip, the migration of leading nucleus, and the distance of nuclear exclusion zone in (**c**). **(e)** Nuclear dynamics in an apical compartment. The images are from Supplementary Video 2. Scale bar, 10 μm. **(f)** Nuclear dynamics in parts of two adjacent subapical compartments. The images are from Supplementary Video 3. Scale bar, 10 μm. The arrows in (**e**,**f**) indicate the direction of hyphal extension. In the experiments, *N. Crassa* strain NMF617 expressing histone H1-RFP is grown in 2.5 mm × 10 μm × 10 μm channels under the constant flow of 10% glucose in 1× Vogels’ salts solution at 0.3 μL/min.

Hyphae of filamentous fungi are composed of multinucleate compartments delimited by porated cross-walls termed septa, and proper movement and positioning of nuclei is crucial to fungal growth and development^7^,^8^,^9^,^38^,^39^. To validate the ability of our system to explore intracellular processes, we monitored the nuclear organization and migration in real-time using the *N. crassa* strain NMF617 with a histone H1-RFP nuclear marker growing in the array of 2.5 mm × 10 μm × 10 μm channels. We first assessed the spatial and temporal distribution of nuclei of 6 hyphae actively growing on a microchip by plotting the number of nuclei per unit length (50 μm) of the hyphae from the conidia to the tips. Figure 4a demonstrates the nuclear distribution of the 6 hyphae growing over the same time period (22 h). The nuclei were approximately uniformly distributed throughout the hyphae, despite the fact that the older distal regions closest to the conidia contained fewer nuclei and the various hyphae ranged in length from 850 to 2450 μm. Moreover, the pattern of distribution and the number of nuclei per unit length in the same hypha slightly changed during hyphal extension (Fig. 4b).

**Figure 5:**
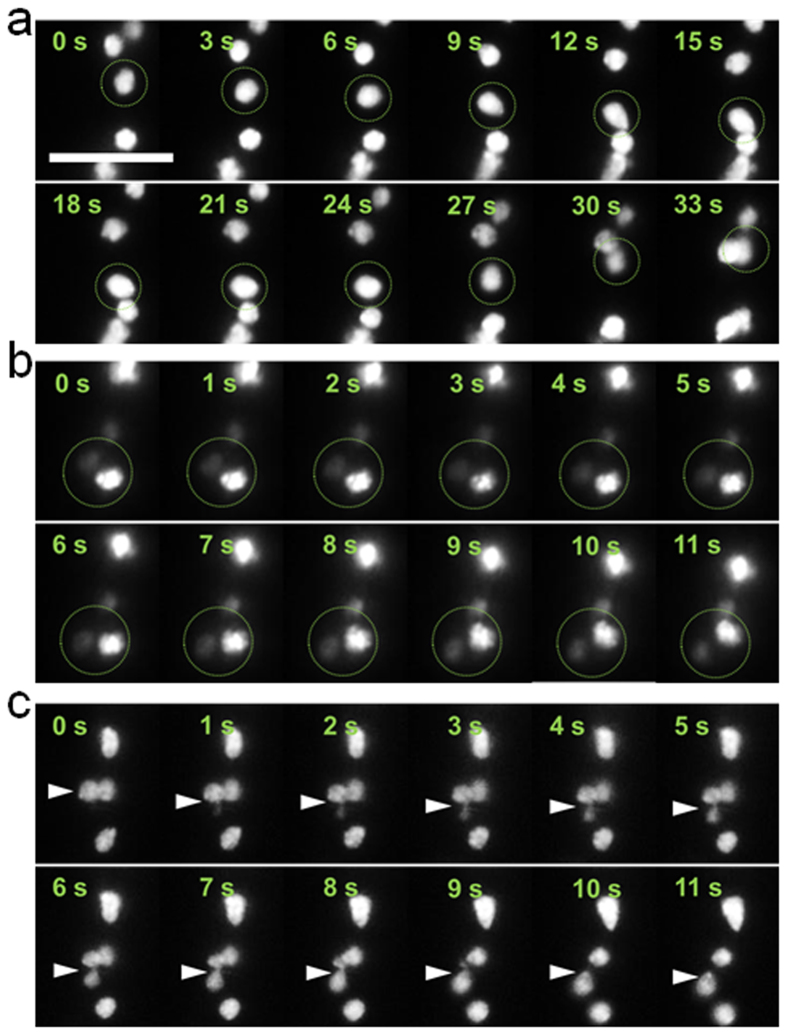
Three major forms of nuclear movement in subapical compartments. (**a**) Oscillation (back-and-forth motion). The circle indicates the oscillated nucleus. **(b)** Bypassing of a fluorescently strong nucleus by a fluorescently weak nucleus. The circle indicates the two nuclei. **(c)** Passage through septa of a nucleus. The arrow indicates the passing nucleus. All images are from Supplementary Video 3. Scale bar, 10 μm. In the experiment, *N. Crassa* strain NMF617 expressing histone H1-RFP is grown in 2.5 mm × 10 μm × 10 μm channels under the constant flow of 10% glucose in 1× Vogels’ salts solution at 0.3 μL/min.

To investigate how the nuclear distribution and organization were maintained, we tested the pattern of nuclear migration in the growing hyphae. For long-range nuclear movement, the apical region of a hypha with an initial length of 955 μm was tracked over 1 h. The nuclei continuously moved towards the tip in concert with the hyphal elongation, while they were always devoid within a certain distance from the apex, forming a nuclear exclusion zone (Fig. 4c). The general forward movement is primarily controlled by cytoplasmic bulk flow, as observed in previous studies^8^,^39^. The migration of the hyphal tip and the leading nucleus (closest to the hyphal apex) were linear over the time period, suggesting that the leading nucleus moved with a velocity (~2 μm/min) similar to that of hyphal tip, and thus the distance of nuclear exclusion zone was approximately constant (14.2 μm on average) (Fig. 4d). To further probe localized nuclear dynamics, we imaged the nuclear migration both in an apical compartment and in parts of two adjacent subapical compartments under high magnification. All nuclei in the apical compartment unidirectionally moved towards the growing tip with oval or pyriform shapes, and some nuclei clustered together (Fig. 4e and Supplementary Video 2). In contrast, the nuclei in the subapical compartments exhibited diverse forms of migration and less elongated shapes (Fig. 4f and Supplementary Video 3). We tracked frequent nuclear motions including irregular oscillation (back-and-forth motion), bypassing of other nuclei, and passing through septa in *N. crassa* (Fig. 5 and Supplementary Video 3), as seen previously in other filamentous fungi without microfluidic confinement^7^. Along with mitosis, these movements contributed to the global even spacing of nuclei along the hyphae, net unidirectional migration and a continuous growth polarity.

### Long-Term Gene Expression Dynamics

**Figure 6:**
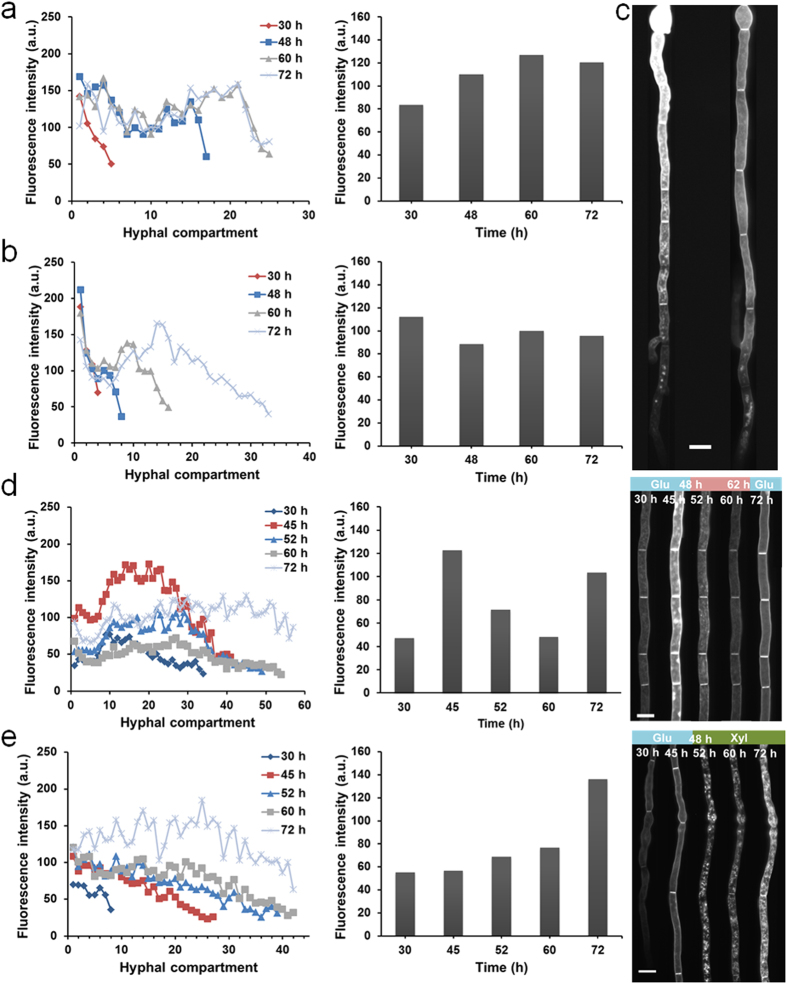
Long-term monitoring of gene expression dynamics on the microfluidic chip. (**a**,**b**) The expression level of xylose transporter-GFP of each hyphal compartment (from conidial end to tip; left panels) and entire hypha (right panels) in two representative hyphae at 30, 48, 60 and 72 h. The hyphae are constantly grown in 10% glucose. **(c)** Two representative hyphae with fluorescent gradient. Scale bar, 10 μm. (**d**,**e**) The expression level of xylose transporter-GFP of each hyphal compartment (from conidial end to tip; left panels) and entire hypha (middle panels) in typical hyphae, and representative images of the same compartments over time (right panels) at 30, 45, 52, 60 and 72 h, in response to **(d)** glucose starvation at 48 h and replenishment at 62 h and **(e)** carbon source switching from 10% glucose (Glu) to 10% xylose (Xyl) at 48 h. Scale bar, 10 μm. In the experiments, *N. Crassa* strain XEB 12 expressing xylose transporter-GFP is grown in 2.5 mm × 10 μm × 10 μm channels under the constant flow of different media at 0.3 μL/min.

Finally, we evaluated the microfluidic device for long-term cultivation and monitoring experiments by detecting the expression of a xylose transporter (NCU00821)-GFP^5^ over 3 days using a *N. crassa* strain XEB 12 in the array of 2.5 mm × 10 μm × 10 μm channels. Sugar transport plays a pivotal role in carbon source metabolism^40^,^41^. Although numerous sugar transporters have been identified and characterized in filamentous fungi, little is known about their spatial and temporal dynamics at the single hypha or even single compartment resolution. We continuously cultivated the strain using glucose as the sole carbon source, and quantified the mean fluorescence intensity of each hyphal compartment at 30, 48, 60 and 72 h (Fig. 6a,b). Overall, the xylose transporter in the compartments of the apical region presented lower expression regardless of the length and growth period of the hyphae, and the GFP transformation reduced the hyphal extension rate as compared to the wild-type strain. The distribution of fluorescence was relatively more uniform in older distal regions. Nevertheless, the majority of transporters in the apical region were concentrated at vacuoles, while very little fluorescence was detected in hyphal periphery. As hyphal extension proceeded, fluorescence intensity in the apical regions increased, whereas the newly synthesized apical regions were still weakly fluorescent. The expression level of xylose transporter in specific compartments varied over the growing process, and the expression pattern presented hypha-to-hypha variability (Fig. 6a,b, left panels). In spite of the different patterns of change seen for each hypha, the mean fluorescence intensity of the entire hyphae did not show a significant increase after 72 h of hyphal growth in the presence of glucose (Fig. 6a,b, right panels), indicating a stable level of expression may have been achieved in response to the local environment. Two representative hyphae with fluorescence gradient along the hyphal extension direction are shown in Fig. 6c.

It is known that *Neurospora* can utilize the pentose sugars, including xylose, as a carbon source using a different pathway from glucose, and xylose transporters are differentially responsive to glucose and xylose^41^. The dynamic regulation of xylose transporter expression by changing environmental conditions was investigated in two conditions: glucose starvation and switching of carbon source from glucose to xylose. Both experiments were stably conducted over 72 hours, and the medium change could be flexibly guided by the microvalves. For the glucose starvation experiment, the hyphae were initially cultivated in 10% glucose for 48 h, then in carbon source-deficient medium for 14 h, and finally back to 10% glucose for another 10 h (Fig. 6d). Following 4 h of glucose deprivation, significant downregulation of xylose transporter expression was detected, and the downregulation continued for another 8 h. After 10 h of glucose replenishment, the fluorescence recovered in a similar pattern as seen before deprivation. While the 72 h image does not show the same intensity as that at 45 h, the expression is higher than the original image at 30 h, indicating a faster upregulation of expression. Interestingly, slight elongation of hyphae occurred during the process of glucose starvation. For the carbon source switching experiment, media was completely exchanged from 10% glucose to 10% xylose at 48 h. Figure 6e demonstrates that the presence of xylose dramatically upregulated the expression of the xylose transporter-GFP fusion after 24 h following media exchange. By comparing the two carbon source shifting experiments, the downregulation of xylose transporter expression by glucose starvation seemed to be a relatively rapid process (4 hours), while an extended period of time (24 hours) was required to achieve an obvious upregulation by xylose. Furthermore, we also noticed that subcellular localization of xylose transporter was changed by media shifting in both experiments. Further work is ongoing to elucidate the underlying mechanism of transporter localization and expression in response to carbon source availability.

## Discussion

Our microfluidic system provides many technological advantages for probing hyphal growth and dynamics. First, it can efficiently trap single conidia and partition each hypha in isolated compartments, ensuring independent and accurate measurement. Second, the media and other components are continuously delivered to hyphae through diffusion, creating equivalent environments for all hyphae. In addition to providing ample nutrients to the growing hyphae, the mode of medium exchange does not perturb the hyphal position, and shear stresses that could impact growth studies are avoided. Third, up to 50 hyphae with limited to no branching can be simultaneously monitored in a semi-automated fashion. Fourth, the layout of the microfluidic platform allows stable and robust long-term operation. Fifth, the temporal and spatial resolution is substantially improved, empowering tracking of hyphal dynamics at the single hypha or even single hyphal compartment level. Together, these technological improvements enable detailed interrogation of cell-to-cell variability/heterogeneity during hyphal growth, distribution and dynamics of intracellular organelles, long-term gene expression dynamics at different developmental stages at specific locations, and cellular response to well-defined environmental signals and chemical stimuli.

The next frontier will come from integrating the improved microfluidic layout with an automated image acquisition microscopy system, faster stage translation motors, higher spatial and temporal resolution optics and detectors, as well as diverse genetically encoded fluorescent molecular markers and selective chemical probes. The platform can also be adapted to probe more complex fungal behaviors such as branching and fusion of different hyphae in a mycelium by creating side channels along a main channel or connecting two main channels. In addition to creating the nutrient concentration gradient by varying the length of growth channels on a single chip as described, the platform can also integrate multiple loading channels into an individual chip to allow comparisons of different fungal strains and studies of interactions (such as dikaryon formation) between fungi of differing genotypes. Besides imaging-based detection, by incorporating a removable cover plate, our microfluidics-based cell culture technique may be combined with laser microdissection to facilitate the precise retrieval of hyphal sections from a target mycelium for downstream molecular biology assays or omics analyses. Such microarray-based transcriptomic studies have been performed at the periphery of fungal colonies cultured with traditional methods by Wösten *et al*.^10^, but when combined with the platform described here would enable targeted analysis of hyphae growing under specific environmental conditions or in the internal regions of a mycelium. However, the current devices and strategies can immediately be extended to a wide variety of other cell types for a broad spectrum of novel investigations revealing the characteristics and mechanisms of long-term polarized cell growth.

## Methods

### Fungal strains and growth conditions

*N. crassa* strains were cultured from –20 °C stocks of conidia on 1× Vogel’s medium N salts^42^ and 1.5% glucose, 1.5% agar slants for 7–10 days before collection with sterile distilled water or 1× Vogel’s salts. Collected conidia were strained through sterile miracloth (EMD Millipore) to remove tissue fragments larger than 20 μm. Histone H1-RFP strain NMF617 was provided as a kind gift by Professor Michael Freitag. Construction of xylose transporter (NCU00821) containing a C-terminal GFP tag was accomplished using a mus-51 mutant strain (FGSC9718) obtained from the Fungal Genetics Stock Center. Transformation of 7–14 day old conidia was done on a BioRad Gene Pulser Xcell Electroporation System in a 2 mm gap cuvette (BioRad) using settings of 1,500 V, 600 Ω, and 25 μF^43^. After a 2 h recovery period in 4 mL Vogel’s salts, the conidia suspension was mixed 1:1 with Vogel’s top agar (2M sorbitol (Sigma-Aldrich), 2× FGS or sorbose (Acros Organics)/fructose (Thermo Fisher Scientific)/glucose (Thermo Fisher Scientific) media, 2% agar, 1× Vogels’ salts), and plated on 15 mL Vogel’s FGS agar containing 2 μg/mL hygromycin B (Life Technologies). Plates were left to grow for 5–7 days at 28 °C, and individual colonies transferred to Vogels’ 1.5% glucose slants. Resulting strains were checked for GFP signal by microscopy and by PCR for correctness. GFP-containing strains were then crossed to FGSC2489 on synthetic crossing medium. Resulting ascospores were spread and germinated (1 h at 65 °C) under on FGS agar containing hygromycin B.

### Microchip fabrication

The polydimethylsiloxane (PDMS)/glass microfluidic chip was constructed using multilayer soft lithography^44^, as described previously with some modifications^45^,^46^. Briefly, the microscale features were designed using AutoCAD software (Autodesk), and reproduced onto photoresist-coated chrome-on-glass photomask blanks (Telic) using an Intelligent Micro Patterning SF-100 Xpress direct-write photolithography system. The mold defining the control layer (25 μm high) was manufactured with a negative photoresist SU-8 25 (Microchem) on a 4-inch silicon wafer (University Wafer). The hybrid mold for the fluidic layer was fabricated on a separate 4-inch silicon wafer in a 3-step process. First, hyphal growth channels, microrulers, mediums infusion channel and parts of conidial trapping sites were made of a negative photoresist SU-8 2010 (Microchem) with 10 μm height. Second, constricted sections of conidial trapping sites were fabricated with another negative photoresist SU-8 2002 (Microchem) with 2 μm height. Third, the conidial loading channel, sample delivery channels, inlets and outlets were created with a positive photoresist AZ P4260 (Capital Scientific) with 10 μm height, followed by thermally reflowing to a rounded cross section at 150 °C for 20 min to allow complete valve closure^44^.

To assemble the microfluidic devices, PDMS prepolymer mixture comprising base monomer and curing agent (Sylgard 184, Dow Corning) at a mass ratio of 10:1 was poured onto the fluidic layer master in a Petri dish to ~5 mm thick, and spin-coated onto the control layer master at 2000 rpm for 30 s to generate the thin membrane (~50 μm thick). After curing both PDMS layers at 70 °C for 1 h, the peeled fluidic layer stamp was precisely aligned with the control layer under a VHX-600 digital microscope (Keyence), and bonded together by plasma oxidation. The two-layer PDMS structure was then baked at 70 °C for another 2 h prior to being peeled off from the control layer master and being punched to generate access holes. Finally, the cured PDMS assembly was covalently bonded onto a pre-cleaned coverslip (Thermo Fisher Scientific) by oxygen plasma treatment to form enclosed channels, followed by baking at 70 °C overnight to enhance bond strength.

### Microfluidic system operation

The cells and reagents were introduced into the microfluidic device through PFA high purity tubing (IDEX Health & Science) driven by a syringe pump (Harvard Apparatus). The pneumatic microvalves were controlled by the application and removal of the pressure *via* external computer-controlled solenoid valve manifolds (Festo). Nitrogen was used as the pressure source, and the pressure was adjusted to 60 psi by a pressure regulator. Custom software developed with C#/.NET 4.5.1 was used to regulate the switching of the solenoid valves. The control channels were pre-filled with water prior to each experiment to prevent introducing bubbles into the fluidic channels.

### Single-conidium trapping and hyphal growth

Trapping and growth conditions were optimized to provide environmental conditions that minimized visible signs of stress (such as vacuolar enlargement) on the growing hyphae. In a typical procedure, all channels were initially primed with 1× Vogels’ salts solution to expel bubbles with the help of pneumatic microvalves. Afterwards, a conidial suspension at a concentration of 2 × 10^5^ cells/mL in 1× Vogels’ salts solution was driven into the device at a flow rate 0.4 μL/min. Following conidial trapping, both the waste outlet valve and medium outlet valve were opened, and a flow of 1× Vogels’ salts solution through the chip washed away any conidia remaining in the channels. For hyphal growth, the isolation valve and the waste outlet valve were actuated, and the 1× Vogels’ salts solution with carbon source was constantly perfused into the medium infusion channel at a flow rate of 0.3 μL/min, corresponding to a velocity of 2.5 mm/s.

### Image acquisition and analysis

The microfluidic chip was mounted on the stage of a PALM MicroBeam inverted fluorescence microscope system (ZEISS) outfitted with 5×, 10×, 20×, 40× and 63× dry objectives, fluorescence filter cubes optimized for GFP and RFP imaging, as well as a AxioCamMRm charge-coupled device (CCD) camera. AxioVision software (ZEISS) was used to acquire fluorescence and bright-field images. For monitoring nuclear dynamics, imaging was conducted using an Axiovert inverted fluorescence microscope (ZEISS) with a 63× oil immersion objective and an electron multiplying charge-coupled device (EMCCD) camera (PhotonMAX; Princeton Instruments). Images and videos were obtained by WinView software (Princeton Instruments). Image processing and analysis were performed using ImageJ software (http://imagej.nih.gov/ij/). Background fluorescence was subtracted from all measured fluorescence intensities.

### Characterization of flow profile and molecular diffusion

Numerical simulations of fluid dynamics and diffusion were performed using commercial finite element modeling software, COMSOL Multiphysics (COMSOL). The basic physics of the simulation was based on incompressible momentum Navier-Stokes equation and Fick’s Law. For flow properties, the density of water, *ρ* = 1000 kg/m^3^, the dynamic viscosity of water, *η* = 8.9 × 10^–4^ Pa·s, and the diffusion coefficient of glucose in water, *D* = 6 × 10^−10^ m^2^/s, were used in the simulations. The velocity in the inlet was set to 2.5 mm/s (0.3 μL/min), and the initial concentration of glucose was set to 555 mol/m^3^ (10%, w/v).

To track molecular diffusion and quantify the concentration profile experimentally, the microfluidic device was first filled with water or grown with *N. crassa* hyphae, and then perfused with 100 μm of fluorescent probe 2-deoxy-2-[(7-nitro-2,1,3-benzoxadiazol-4-yl)amino]-D-glucose (2-NBDG; Sigma-Aldrich) solution into the medium infusion channel using a syringe pump. Fluorescence images of hyphal growth channels were captured at variable time points after medium introduction, and intensity was quantified with ImageJ software.

## Additional Information

**How to cite this article**: Geng, T. *et al*. Compartmentalized microchannel array for high-throughput analysis of single cell polarized growth and dynamics. *Sci. Rep*. **5**, 16111; doi: 10.1038/srep16111 (2015).

## Supplementary Material

Supplementary Information

Supplementary Video S1

Supplementary Video S2

Supplementary Video S3
