# Supplementary material for: Compartmentalized microchannel array for high-throughput analysis of single cell polarized growth and dynamics
Source: Sci Rep. 2015 Nov 4;5:16111. doi: 10.1038/srep16111 (PMC4632079; doi:10.1038/srep16111)
Supplement: Supplementary Information [file srep16111-s1.pdf]

# Supplementary information

# Compartmentalized microchannel array for high-throughput analysis of single cell polarized growth and dynamics

Tao Geng, Erin L. Bredeweg, Craig J. Szymanski, Bingwen Liu, Scott E. Baker, Galya Orr, James E. Evans, and Ryan T. Kelly

Environmental Molecular Sciences Laboratory, Pacific Northwest National Laboratory, Richland, WA, USA.

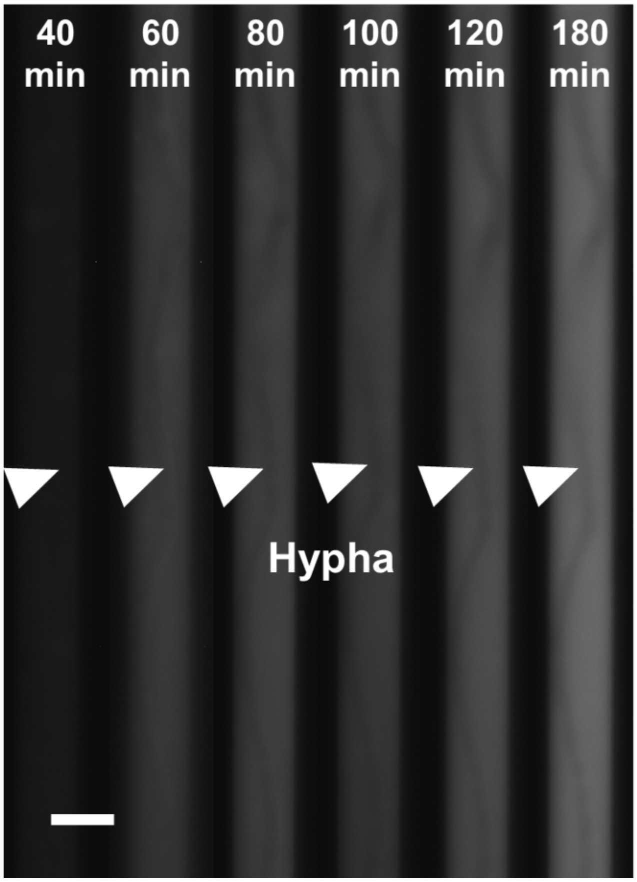


**Supplementary Figure 1.** Diffusion of 2-NBDG into a channel filled with a growing hypha. The fluorescence intensity increases over time, indicating medium exchange is not blocked in the hyphal growth channels (2.5 mm × 10 μm × 10 μm) by the hyphal extension. Scale bar, 10 μm.

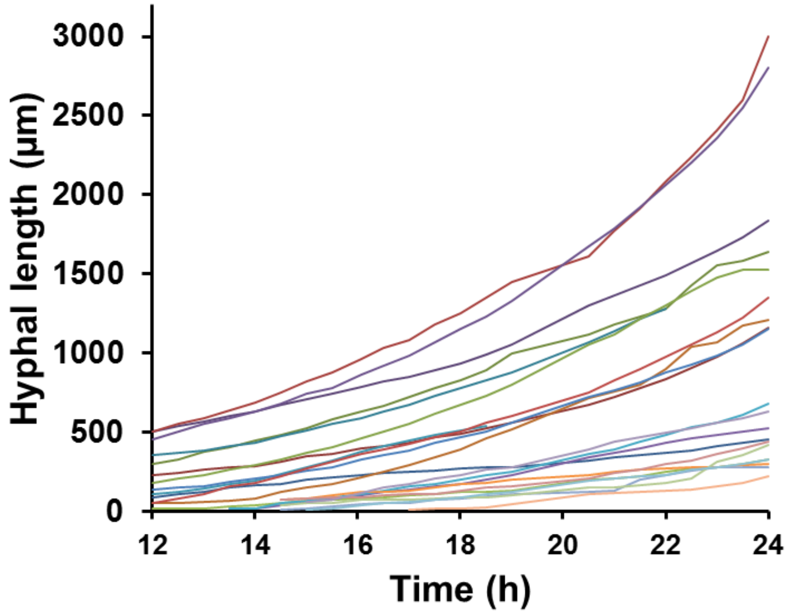


**Supplementary Figure 2.** Growth curves of 27 individual hyphae growing in the channels (2.5 mm × 10 µm × 10 µm) on a single microchip. In the experiment, wild-type *N. Crassa* is grown under the constant flow of 10% glucose in 1× Vogels' salts solution at 0.3 µL/min.

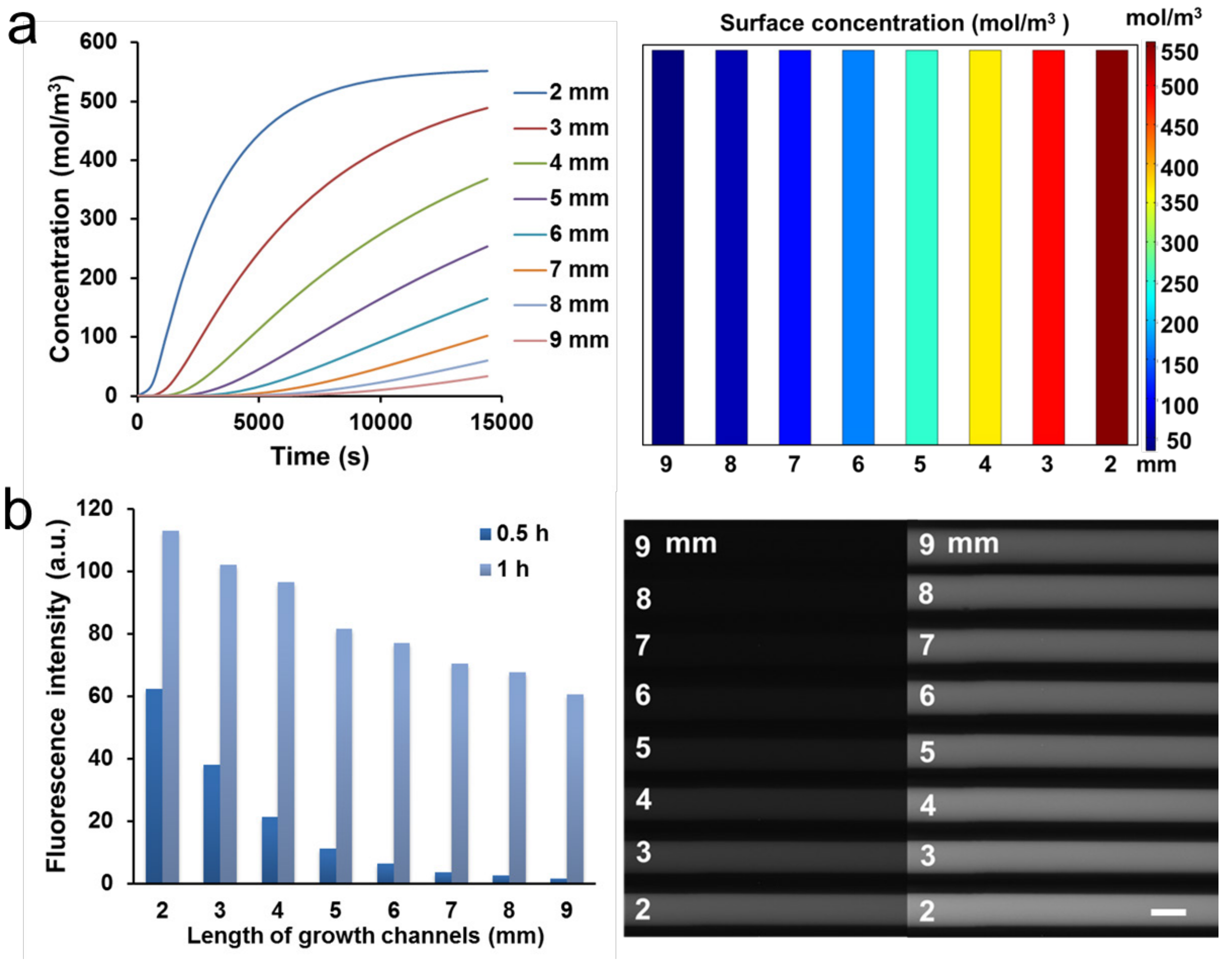


**Supplementary Figure 3.** Chemical concentration gradient on the microfluidic chip with different lengths of channels. **(a)** Numerical simulation of time-dependent diffusion of glucose in the hyphal growth channels with lengths of 2, 3, 4, 5, 6, 7, 8 and 9 mm using COMSOL Multiphysics software. The initial concentration of the glucose is 555 mol/m$^3$ (10% w/v). Right panel shows the glucose concentration in the 120 µm of regions from the trapping sites after 4 h of diffusion. **(b)** Visualization and quantification of the gradient profile after flowing 100 µM of 2-NBDG solution for 0.5 h and 1 h. Scale bar, 10 µm.

**Supplementary Video 1.** Hyphal extension of *N. crassa* histone H1-RFP strain NMF617 in a 2.5 mm × 10 µm × 10 µm growth channel. The video is taken for 5 min at intervals of 1 s.

**Supplementary Video 2.** Nuclear dynamics in an apical compartment of *N. crassa* histone H1-RFP strain NMF617 in a 2.5 mm × 10 µm × 10 µm growth channel. All nuclei unidirectionally move towards the growing tip. The video is taken for 5 min at intervals of 1 s.

**Supplementary Video 3.** Nuclear dynamics in parts of two adjacent subapical compartments of *N. crassa* histone H1-RFP strain NMF617 in a 2.5 mm × 10 µm × 10 µm growth channel. The nuclei move irregularly including oscillation, bypassing and passing through septa. The video is taken for 5 min at intervals of 1 s.
